# New Procedure in the Operation for Phimosis

**Published:** 1881-03

**Authors:** 


New Procedure in the Operation for Phimosis, R. J. Levis, M.D.—A new instrument. The object of the instrument is to facilitate the entire excision of the inner inelastic mucous membrane of the prepuce, without removing any, or more than may be required, of the outer normal skin. In general form it somewhat resembles the ordinary mathematical compasses or dividers. The limbs, or blades, terminate in blunt points, and are deeply serrated on their outer surfaces, with points or teeth set backward, like fine saw-teeth, for the purpose of firmly holding the mucous membrane without the risk of slipping when traction is made. The blades are forced apart by a thumb-screw. A cut of the instrument is given in *New York Medical Record.*

